# The Euroflow PID Orientation Tube in the diagnostic workup of primary immunodeficiency: Daily practice performance in a tertiary university hospital

**DOI:** 10.3389/fimmu.2022.937738

**Published:** 2022-09-13

**Authors:** Jana Neirinck, Annelies Emmaneel, Malicorne Buysse, Jan Philippé, Sofie Van Gassen, Yvan Saeys, Xavier Bossuyt, Stefanie De Buyser, Mirjam van der Burg, Martín Pérez-Andrés, Alberto Orfao, Jacques J. M. van Dongen, Bart N. Lambrecht, Tessa Kerre, Mattias Hofmans, Filomeen Haerynck, Carolien Bonroy

**Affiliations:** ^1^ Department of Diagnostic Sciences, Ghent University, Ghent, Belgium; ^2^ Department of Laboratory Medicine, Ghent University Hospital, Ghent, Belgium; ^3^ Data Mining and Modelling for Biomedicine Group, Vlaams Instituut voor Biotechnologie (VIB) Center for Inflammation Research, Ghent, Belgium; ^4^ Department of Applied Mathematics, Computer Science and Statistics, Ghent University, Ghent, Belgium; ^5^ Department of Microbiology, Immunology and Transplantation, KU Leuven, Leuven, Belgium; ^6^ Department of Laboratory Medicine, KU Leuven University Hospitals Leuven, Leuven, Belgium; ^7^ Department of Public Health and Primary Care, Ghent University, Ghent, Belgium; ^8^ Laboratory for Pediatric Immunology, Department of Pediatrics, Leiden University Medical Center, Leiden, Netherlands; ^9^ Cancer Research Centre (Instituto de Biología Molecular y Celular del Cáncer (IBMCC), USAL-CSIC; CIBERONC CB16/12/00400), Institute for Biomedical Research of Salamanca (IBSAL), Department of Medicine and Cytometry Service (NUCLEUS Research Support Platform), University of Salamanca (USAL), Salamanca, Spain; ^10^ Translational and Clinical Research Program, Centro de Investigación del Cáncer and Instituto de Biología Molecular y Celular del Cáncer, Consejo Superior de Investigaciones Científicas (CSIC)-University of Salamanca (USAL), Department of Medicine, IBSAL and Centro de Investigación Biomédica en Red de Cáncer (CIBERONC), University of Salamanca, Salamanca, Spain; ^11^ Department of Immunology, Leiden University Medical Cente, Leiden, Netherlands; ^12^ Laboratory of Mucosal Immunology, VIB-UGhent Center for Inflammation Research, Ghent University, Ghent, Belgium; ^13^ Department of Internal Medicine and Pediatrics, Faculty of Medicine and Health Sciences, Ghent University, Ghent, Belgium; ^14^ Department of Pulmonary Medicine, University Hospital Ghent, Ghent, Belgium; ^15^ Department of Hematology, Ghent University Hospital, Ghent, Belgium; ^16^ Department of Pediatric Pulmonology and Immunology and Primary Immunodeficiency (PID) Research Lab, Ghent University Hospital, Ghent, Belgium

**Keywords:** flow cytometry, immunophenotyping analysis, EuroFlow standardization, clinical validation, primary immunodeficiencies (PID)

## Abstract

**Introduction:**

Multiparameter flow cytometry (FCM) immunophenotyping is an important tool in the diagnostic screening and classification of primary immunodeficiencies (PIDs). The EuroFlow Consortium recently developed the PID Orientation Tube (PIDOT) as a universal screening tool to identify lymphoid-PID in suspicious patients. Although PIDOT can identify different lymphoid-PIDs with high sensitivity, clinical validation in a broad spectrum of patients with suspicion of PID is missing. In this study, we investigated the diagnostic performance of PIDOT, as part of the EuroFlow diagnostic screening algorithm for lymphoid-PID, in a daily practice at a tertiary reference center for PID.

**Methods:**

PIDOT was tested in 887 consecutive patients suspicious of PID at the Ghent University Hospital, Belgium. Patients were classified into distinct subgroups of lymphoid-PID vs. non-PID disease controls (non-PID DCs), according to the IUIS and ESID criteria. For the clinical validation of PIDOT, comprehensive characterization of the lymphoid defects was performed, together with the identification of the most discriminative cell subsets to distinguish lymphoid-PID from non-PID DCs. Next, a decision-tree algorithm was designed to guide subsequent FCM analyses.

**Results:**

The mean number of lymphoid defects detected by PIDOT in blood was 2.87 times higher in lymphoid-PID patients vs. non-PID DCs (p < 0.001), resulting in an overall sensitivity and specificity of 87% and 62% to detect severe combined immunodeficiency (SCID), combined immunodeficiency with associated or syndromic features (CID), immune dysregulation disorder (ID), and common variable immunodeficiency (CVID). The most discriminative populations were total memory and switched memory B cells, total T cells, TCD4+cells, and naive TCD4+cells, together with serum immunoglobulin levels. Based on these findings, a decision-tree algorithm was designed to guide further FCM analyses, which resulted in an overall sensitivity and specificity for all lymphoid-PIDs of 86% and 82%, respectively.

**Conclusion:**

Altogether, our findings confirm that PIDOT is a powerful tool for the diagnostic screening of lymphoid-PID, particularly to discriminate (S)CID, ID, and CVID patients from other patients suspicious of PID. The combination of PIDOT and serum immunoglobulin levels provides an efficient guide for further immunophenotypic FCM analyses, complementary to functional and genetic assays, for accurate PID diagnostics.

## 1 Introduction

Primary immune deficiency diseases (PIDs) comprise rare and frequently life-threatening inherited disorders with defects in one or multiple components of the innate and/or adaptive immune system. These impairments lead to a wide spectrum of clinical manifestations such as severe and/or recurrent infections, autoimmunity, polyclonal immune cell proliferation or malignancies, and immunophenotypic aberrancies. Severe combined immunodeficiency (SCID) is caused by a major T-cell maturation defect, often associated with B-cell and/or natural killer (NK) cell defects resulting in life-threatening infections (1). Combined immunodeficiency (CID) is characterized by a variable immunophenotype ranging from normal to multiple aberrant T-cell subsets and a heterogeneous clinical picture often with associated and/or syndromic features (1). Primary antibody deficiency (PAD), including common variable immunodeficiency (CVID), manifests with recurrent infections, hypogammaglobulinemia, and poor response to vaccination (1, 2).

Early diagnosis for rapid initiation of appropriate treatment is of utmost importance in all PIDs, to improve patient outcome (3–5). This is particularly true for SCID, where early diagnosis and treatment, before occurrence of life-threatening infections and other disease complications, positively impacts overall survival rates (90%–95% in patients diagnosed early vs. 81%–82% in patients with late diagnosis) (6, 7). In case of CVID, life-long immunoglobulin (Ig) replacement therapy (IGRT) also improves quality of life and decreases the severity and frequency of infections. Moreover, CVID patients with non-infectious complications have a poor long-term prognosis (40-year overall survival rate of 42% in CVID patients with complications compared to 95% in those without) (8), which may be related to delayed diagnosis and delayed IGRT, as this treatment also impacts some of the disease-related non-infectious clinical manifestations and/or complications (9).

Well-established guidelines to recognize PID suspicion (‘the internationally validated 10 PID warning signs’) as well as widely accepted clinical diagnostic/classification criteria for PID have been proposed (1, 10–13). Besides careful documentation of personal and family history (infections, auto-inflammation, autoimmunity, and malignancies) together with physical examination, basic immunological screening tests are critical in the early PID diagnostic workup. Among others, mandatory laboratory tests include white blood cell (WBC) count and differentiation, quantitation of serum Ig (sIg) isotypes and IgG subclasses, and antibody-based immune response to specific antigens—e.g., response to both protein-based (e.g., tetanus) and unconjugated polysaccharide pneumococcal vaccine—together with multiparameter flow cytometry (FCM) immunophenotyping. These laboratory tests provide a first glance on the immune system, aimed to guide further decisions on the need for more specialized assays such as disease-specific protein quantification, functional assays (e.g., T-cell proliferation), signaling pathway analysis (e.g., STAT phosphorylation), and importantly, genetic analysis (next-generation sequencing/whole-exome sequencing), for identification of both variants in known PID-associated genes and new PID-causing genetic defects (11, 14, 15).

Currently, multiparameter FCM analyses can be positioned both at an early phase of the PID diagnostic workup, for enumeration and characterization of immune cell composition in blood and guidance for subsequent genetic analyses, and/or after genetic analyses have been performed, for confirmation and interpretation of the genetic (e.g., TREC screening) results, making both techniques complementary (15, 16). Among other advantages, FCM is a fast (<24 h to results), widely available, and (relatively) affordable technique, which contrasts with the longer turnaround time and higher costs associated with genetic analyses (15).

Despite its critical role in PID diagnosis and classification, multiparameter FCM still faces several challenges, mainly in terms of standardization, the availability of (appropriate) age-matched reference values, and expert-dependent (subjective) data analysis and interpretation. In this context, the EuroFlow Consortium has recently developed and validated a fully standardized multiparameter FCM immunophenotyping an “all-in-one” pipeline, comprising standard operating procedures (SOPs) for instrument setup and calibration and for sample preparation, reagent panels, (pre)analytical, and postanalytical data analysis tools and procedures. These standardization efforts made it possible to generate reliable and reproducible results across different instruments, laboratories, and countries, which are further supported through the EuroFlow Quality Assessment (QA) Program (17–20) and continuous updates alongside the fast innovation in the field of flow cytometry (www.EuroFlow.org) (16, 21–31). In this regard, the EuroFlow Consortium has also proposed recently a new FCM-based diagnostic screening and classification algorithm for lymphoid-PID, based on multiple eight– to 12-color single-tube antibody combinations (30). Among all proposed tubes, PID Orientation Tube (PIDOT) emerges as a universal screening tool for the diagnosis of lymphoid-PID in cases suspicious of PID (31). This tube, in combination with the EuroFlow PIDOT reference database, including age-matched reference ranges, allows identification and interpretation of >20 different leucocyte populations, including 15 T-, B-, and NK-cell populations, in peripheral blood. PIDOT has been technically validated on 99 PID patients with defined genetic lesions, and a software tool for automated, expert-guided gating and identification is available (31–33). According to the EuroFlow PID algorithm, both the clinical presentation and PIDOT help in guiding for subsequent more detailed B- and T-cell maturation analyses and complementary functional and genetic assays, in a highly efficient way (15, 25, 30, 34, 35). Of note, more detailed FCM analyses increase insight in PID classification, since associations between defective lymphoid patterns and clinical features are well established (35, 36).

In this study, we investigated the clinical utility of PIDOT in a real-world tertiary hospital-based setting, *via* analysis of a large series of 887 consecutive patients suspected to have PID. Clinical validation of PIDOT on such a wider spectrum of patients has not yet been reported. We performed a comprehensive characterization of lymphoid defects identified with PIDOT, to determine the most discriminative cell populations to distinguish lymphoid-PID from non-PID disease controls. In addition, we designed an optimized decision-tree algorithm to guide subsequent more extensive FCM analyses and to support an FCM-based lymphoid-PID classification.

## 2 Materials and methods

### 2.1 Study design and patient population

**Figure 1:**
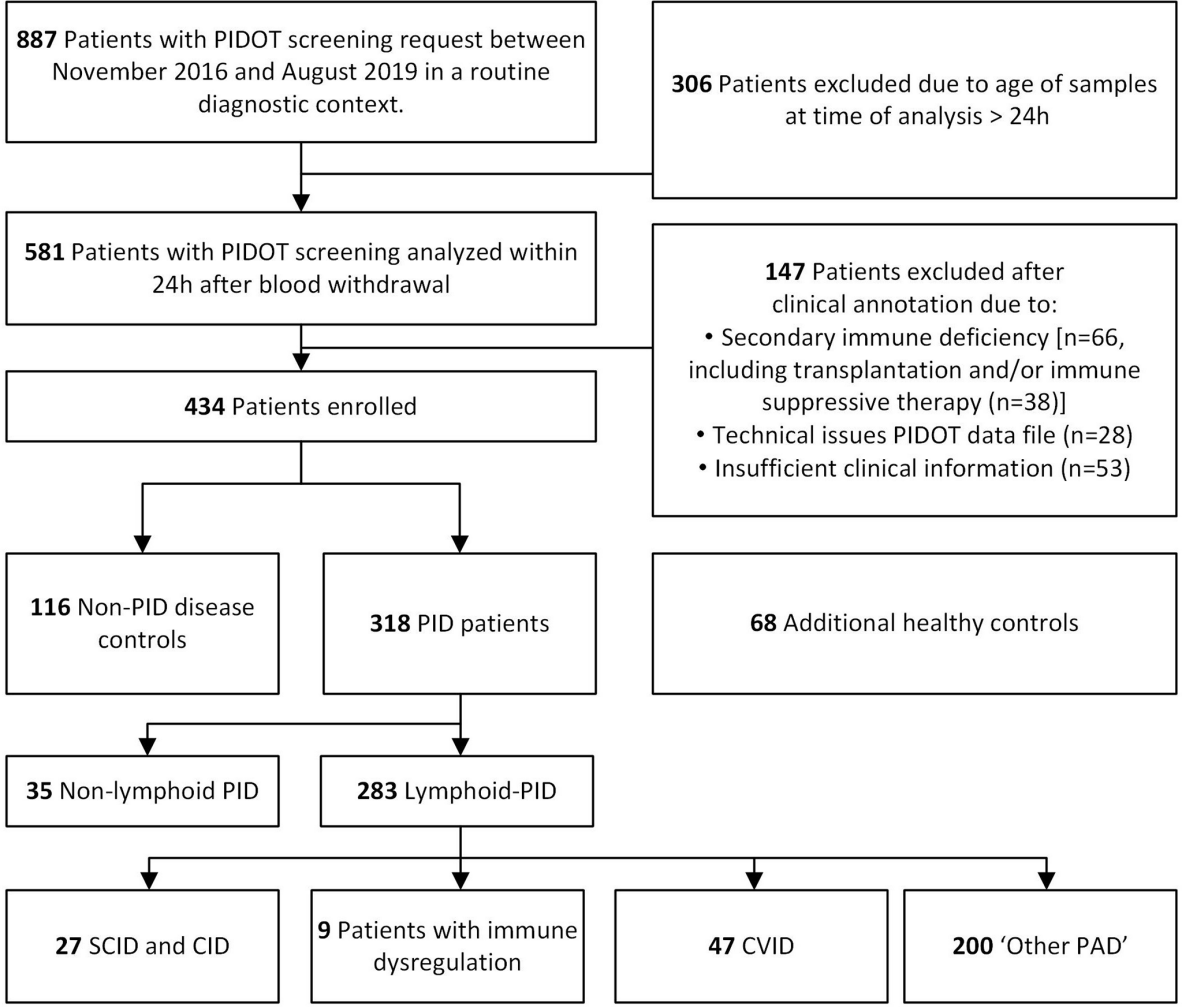
Flowchart diagram of study design and population. Blood samples of patients suspected to have primary immunodeficiency (PID) were analyzed with PID Orientation Tube (PIDOT). After exclusion based on clinical annotation and flow cytometric data quality, patients were classified in lymphoid-PID subgroups [including (severe) combined immunodeficiencies [(S)CID], immune dysregulation disorders (ID), common variable immunodeficiency (CVID), and other primary antibody deficiencies (PAD), non-PID disease controls, and non-lymphoid-PID patients.

PIDOT was stained on peripheral blood (PB) samples, collected from 887 patients with PID suspicion, between November 2016 and August 2019, at the Departments of Paediatrics, Haematology and Pneumology, Center for Primary Immunodeficiency Ghent, Jeffrey Modell Diagnosis and Research Center, at the Ghent University Hospital, Belgium. PB samples were preferably collected beyond acute infectious episodes. An overview of the patient inclusion criteria is shown in **Figure 1**. Samples older than 24 h (n = 306) and samples from patients with secondary immunodeficiencies (n = 66), with missing clinical information or with FCM data files of insufficient quality (n = 81) at time of PIDOT analysis, were excluded (**Supplementary Table 1**) (37). Finally, a study population of 434 patients was retained and classified in (a) patients with PID diagnosis (clinically and/or genetically defined, according to ESID and IUIS criteria [lymphoid-PID (n = 283) or non-lymphoid-PID (n = 35)]) and (b) non-PID disease controls (non-PID DCs, n = 116) in whom PID diagnosis was ruled out (as defined by the treating physician based on standard clinical care) (1, 12, 38). Patients with features of both non-lymphoid-PID and lymphoid-PID were categorized either as non-lymphoid-PID or lymphoid-PID based on the most prominent immunological features and/or underlying genetic defect. In parallel, 68 healthy controls (HCs), both children and adults, without signs or suspicion of immunological (no history of recurrent/severe infections and allergy) or hematological diseases, were recruited and analyzed with the PIDOT for verification of the age-matched reference ranges previously defined by the EuroFlow Consortium (31). Within this healthy control cohort, most age categories as defined in the EuroFlow reference database were represented (5 to 20 HCs per age category), except for the ‘cord blood’, ‘newborn’, and ‘above 70 years’ categories. The age categories between 1 and 23 months were pooled into one single age category to obtain a sufficient series.

### 2.2 Basic immunological screening

Basic immunological screening was performed at time of PIDOT analysis, as part of the PID diagnostic workup. Serum Ig (sIg) concentrations were measured on fresh serum samples on a Roche Cobas 8000A c502 (Roche Diagnostics NV, Basel, Switzerland) and a Behring Nephelometer Analyzer II (Siemens Healthcare Diagnostics NV, Erlangen, Germany) for IgG, IgA, and IgM, and IgG_2_ and IgG_3_. The absolute WBC and lymphocyte counts were determined on K_2_EDTA whole blood using a Sysmex XE-5000 hematology analyzer (Sysmex Corporation, Kobe, Japan). For statistical purposes, the sIg measurements were converted into categorical data (above or below the mean ± 2 standard deviations [SD]) and z-scores by evaluation against the established reference values (39, 40). For each patient, information on immunoglobulin replacement therapy (IGRT) at time of basic immunological screening was recorded (**Supplementary Table 2**).

### 2.3 EuroFlow-based flow cytometry immunophenotyping

Patients with PID suspicion (n = 434) and healthy controls (n = 68) were screened with PID Orientation Tube (PIDOT), strictly following the EuroFlow SOPs available at www.EuroFlow.org (19, 27). For manual data analyses, the Infinicyt™ software (version 1.8 - 2.0.4b; Cytognos SL, Salamanca, Spain) was used following the EuroFlow manual gating strategy focused on the lymphoid populations (30–32). The absolute lymphocyte cell counts (/µL) were calculated for each identified lymphocyte population (n = 21) and were compared, together with % of lymphocytes, to the EuroFlow age-matched p5-p95 reference ranges to identify lymphoid abnormalities in each patient. To adjust for age, absolute cell counts (/µL) per lymphocyte (sub)population were converted into categorical data based on reference percentile ranges (31, 32). More details regarding the EuroFlow staining procedures, the instrument setup and calibration, data acquisition, gating strategy, and calculations are described in **Supplementary Methods** and **Supplementary Table 3**.

### 2.4 Design of a decision-tree algorithm

The input dataset for decision-tree modeling consisted of 24 FCM-based PIDOT features supplemented with age-, sex-, and age-adjusted sIg levels (including IgG, IgG_2_, IgG_3_, IgA, and IgM, in case no IGRT at time of basic immunological screening). The 24 FCM-based PIDOT features included absolute cell counts (/µL) per lymphocyte population converted into categorical data (n = 21), supplemented with the lymphocytes (% of WBC) and the % of total memory and switched memory B cells (expressed on total B cells and limited to patients above the age of 4 years)] (12, 41, 42). The non-lymphoid-PID patient samples were not considered in the input dataset but were used to challenge the final proposed decision-tree algorithm. Detailed description of the design of the proposed decision-tree algorithm can be found in **Supplementary Methods**. In brief, supervised machine learning was performed to select the most predictive features distinguishing between lymphoid-PID (groups) and non-PID disease controls using Recursive Partitioning (“rpart” package) in R followed by 10-fold cross-validation (43). Next, the most predictive features were used as input features in ‘rpart’ to design the final proposed decision-tree algorithm. Afterward, the final proposed decision-tree algorithm was compared with the previously published EuroFlow PID screening and classification algorithm for guiding more detailed FCM-based analyses.

### 2.5 Statistical methods

Statistical analyses and graphical representations were performed using either SPSS (version 27.0.1.0, IBM, Armonk, NY), R (version 4.0.3; https://www.r-projects.org), or MedCalc statistical software (version 12.3.0.0, MedCalc Software bvba, Ostend, Belgium) (44, 45). Conversion of continuous data into age-adjusted categorical data was performed using Microsoft Excel for windows (version 2109, Microsoft, Redmond, WA). Count data were compared using negative binomial regression models (46). Continuous variables were compared using the Kruskal–Wallis rank-sum test followed by *post-hoc* comparison using the Mann–Whitney test. Proportions were compared using the chi-squared test. The reported unadjusted p-values were compared against a Bonferroni-adjusted significance level (α = 0.05/k with k = number of pairwise comparisons). Performance of PIDOT and the decision-tree algorithm was assessed based on sensitivity, specificity, positive likelihood ratios (LR+), and cross-validated area under the curve (cvAUC) of the receiver operating characteristic (ROC) curve (47). More details of the statistical methods applied can be found in **Supplementary Methods**.

## 3 Results

### 3.1 Study population characteristics

The study population included lymphoid-PID (n = 283), non-lymphoid-PID (n = 35) patients, and non-PID disease controls (non-PID DCs) (n = 116). The median age of PID (both lymphoid and non-lymphoid) patients was 14 years (range: 0.1–81 years; M:F ratio of 0.92), while non-PID DCs had a median age of 2.4 years (range: 0.1–72 years, M:F ratio of 1.7) (**Supplementary Table 2**).

The lymphoid-PID patients (n = 283) were divided into four PID groups based on the IUIS and ESID classification: 1) 27 patients had SCID or CID with associated and/or syndromic features; 2) 47 PAD patients were diagnosed with clinical CVID, of which 42 had been diagnosed based on the ESID criteria (including decreased IgG/A and poor vaccination response and/or low switched memory B cells with exclusion of secondary causes of immunodeficiency), four patients with a CVID-like phenotype [IKAROS deficiency (n = 2) and activated PI_3_ kinase delta syndrome (APDS, n = 2)], and one patient with X-linked agammaglobulinemia (XLA); 3) 200 cases had another PAD (‘Other PAD’); and 4) nine patients were diagnosed with an immune dysregulation (ID) disorder, including patients with autoimmune lymphoproliferative syndrome (ALPS) (**Figure 1** and **Table 1**). The ‘other PAD’ cohort included patients with isolated IgG subclass deficiency (n = 21), transient hypogammaglobulinemia of infancy (THI, n = 7), selective IgA or IgM deficiency (n = 2 and n = 1, respectively), specific antibody deficiency (SPAD, n = 31), class switch recombination defects and hyper-IgM syndrome (n = 1), and unclassified idiopathic primary hypogammaglobulinemia (n = 137) (**Table 1**). The clinical characteristics of the non-PID DCs can be found in **Supplementary Table 4**.

**Table 1:** PID diagnoses of the study population.

PID diagnoses	Patients, *n*
**SCID and CID with associated or syndromic features**	27
T-B-SCID (*ADA*) Cartilage Hair syndrome Partial DiGeorge syndrome (*22q11* del) WAS (*WAS*) ATM (*ATM)* CHARGE syndrome *(CHD7)* Roifman syndrome (*RNU4ATAC)* Bloom syndrome (*BLM)* THES (*TTC37, SKIV2L)* Wiedemann–Steiner syndrome *(KMT2A)* Kabuki syndrome *(KMT2D)* HIES *(ILST6)* Undefined CID	1 1 6 1 4 1 2 1 2 2 1 3 2
**Predominantly antibody deficiency (PAD)**	**247**
**CVID**	**47**
CVID XLA (*BTK*) IKAROS (*IKZF1*) APDS (*PIK3CD)*	42 1 2 2
**Other PAD**	**200**
SPAD^$^ IgG subclass deficiency Selective IgA deficiency THI Selective IgM deficiency CSR defects and HIGM syndrome Unclassified PAD	31 21 2 7 1 1 137
**Diseases of immune dysregulation**	**9**
ALPS Unclassified disorder of immune dysregulation	7 2
**Non-lymphoid PID**	**35**
MBL deficiency (*MBL*) Complement deficiency (*C3, C2*, *CFI*) Barth syndrome (*TAZ*) TRAPS (*TNFRSF1A*) X- CGD (*CYBB)* IRAK4 deficiency (*IRAK4*) Shwachman–Diamond syndrome (*SBDS*) Muckle–Wells syndrome (*NLRP3)* Clericuzio syndrome (*USB1*) FMF (*MEFV*) AR CGD (*NCF1*) Unclassified innate immunodeficiency Unclassified Phagocytic disorder	11 4 1 2 1 1 2 1 1 1 1 7 2
**Non-PID disease controls**	**116**

^$^SPAD was diagnosed after assessment of the anti-pneumococcal polysaccharide antibodies IgG type 8, 9N, and 15B pre- and post-PPV-23 vaccination, based on the Orange et al. (48) criteria. CID, combined immunodeficiencies; PAD, predominantly antibody deficiencies; SCID, severe combined immunodeficiency; WAS, Wiskott–Aldrich syndrome; ATM, ataxia-telangiectasia; THES, Tricho-hepato-enteric syndrome; HIES, hyper IgE-syndrome; XLA, X-linked agammaglobulinemia; APDS, activated p110δ syndrome; SPAD, specific IgG deficiency; THI, transient hypogammaglobulinemia of infancy; CSR, class switch recombination; HIGM, hyper IgM; ALPS, autoimmune lymphoproliferative syndrome; MBL, mannose-binding lectin; TRAPS, TNF receptor-associated periodic syndrome; X-CGD, X-linked chronic granulomatous disease; FMF, familial Mediterranean fever; AR, autosomal recessive.

As expected from the clinical definition, age-adjusted sIg levels (IgG, IgA, and IgG subclasses) were significantly lower in PAD (both ‘CVID’ and ‘other PAD’) compared to non-PID DCs and non-lymphoid-PID patients (p < 0.001) (**Supplementary Figure 1**). Also, the switched memory B cells (% of total B cells) were significantly lower in CVID compared to non-PID DCs (p < 0.001) (**Supplementary Figure 2**).

### 3.2 Verification of the EuroFlow reference database

The verification of the EuroFlow reference database within the independent group of 68 healthy controls is presented in **Supplementary Table 5**. Overall, 1.8% and 3.5% of observations (n = 1,360, 20 cell populations in 68 samples) scored below p5 and above p95, respectively, making the application of the EuroFlow manual gating approach and reference database suitable for routine use in our laboratory and in this study.

### 3.3 Comprehensive characterization of lymphoid defects detected with PIDOT

#### 3.3.1 Frequency of altered lymphocyte population counts

**Figure 2:**
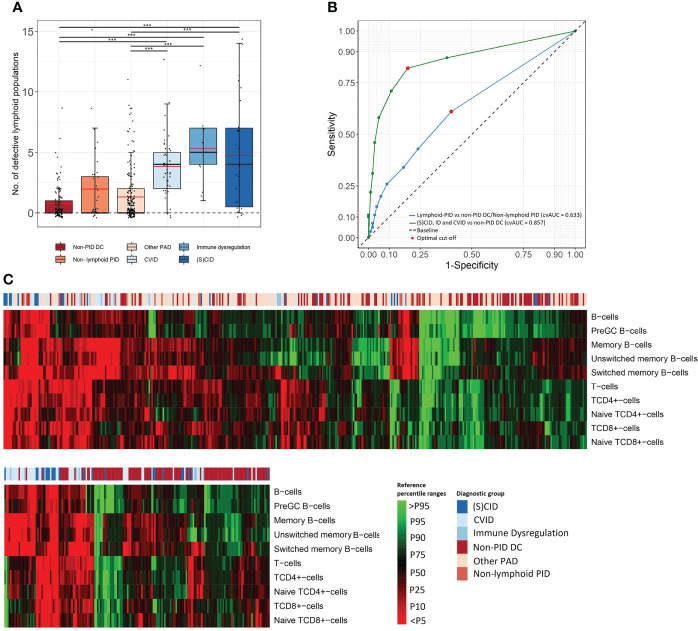
Comprehensive characterization of lymphoid defects. **(A)** Box plots present frequency of total defective lymphoid populations (over the 22 FCM PIDOT variables). The boundaries of the box plots represent the 25th and 75th percentiles. Black and red lines indicate the median and mean, respectively. ***p < 0.001, Mann–Whitney rank-sum test. **(B)** The receiver operating characteristic (ROC) curve and 10-fold cross-validated Area Under the Curve (cvAUC) to assess performance of PIDOT in relation to the total number of lymphoid defects to distinguish lymphoid-PID against a background of non-PID disease controls and non-lymphoid-PID patients (blue ROC curve) and to distinguish (S)CID, ID, and CVID patients from non-PID disease controls (green ROC curve). The optimal cutoff is presented as red dots. **(C)** Heatmap on individual patient level of lymphoid populations of the total study population (top) and (S)CID, ID, and CVID patients vs. non-PID DCs (bottom) as obtained by hierarchical clustering. Non-PID DC, non-PID disease controls; PAD, predominant antibody deficiency; CID, combined immunodeficiency; CVID, common variable immunodeficiency; SCID, severe combined immunodeficiency; ID: immune dysregulation.

A comprehensive characterization of all lymphoid defects identified in our cohort was performed. The total number of lymphoid defects (defined as cell counts below p5 of the corresponding age-matched EuroFlow reference ranges) per patient in each (PID) diagnostic cohort is shown in **Figure 2A** and **Supplementary Figure 3**. The mean number of defective lymphoid populations was 2.87 times higher in lymphoid-PID (n = 283) compared to non-PID DCs (n = 116) (**Table 2**). The sensitivity of the total number of lymphoid defects to identify lymphoid-PID against a background of non-PID DCs and non-lymphoid-PID patients was 61% with a LR+ of 1.55 at the optimal cutoff [defined as ≥1 lymphoid subpopulation defect(s)] (**Table 2** and **Figure 2B**). Similarly, the (S)CID, ID, and CVID patients showed a mean number of lymphoid defects being 6.26, 6.72, and 5.01 times higher compared to non-PID DCs, respectively (**Table 2**). Moreover, within the PAD group, the mean number of lymphoid defects was 2.89 (95% CI: 1.97–4.32) times higher in CVID compared to ‘other PAD’ (p < 0.001), and the latter did not significantly differ from non-PID DCs. The sensitivity of the total number of lymphoid defects to discriminate (S)CID, ID, and CVID patients (n = 83) from non-PID DCs was 87% with a LR+ of 2.29 (**Figure 2B**). However, based on ROC analysis (**Figure 2B**), a cutoff of ≥2 lymphoid defect(s) was found more optimal to differentiate (S)CID, ID, and CVID patients from non-PID DCs (sensitivity = 82%, LR+ = 4.32). More details on cvAUC, sensitivity, specificity, and LR+ for each lymphoid-PID cohort are shown in **Figure 2B** and **Table 2**. In the non-PID DC group, 38% of the samples showed one or more lymphoid defect(s). For most (n = 15/22, 68%) lymphocyte populations, less than 5% of the patients showed decreased cell counts in blood, while the number of populations with increased cell counts above p95 was comparable among the different patient groups (**Supplementary Figure 4**).

**Table 2:** Sensitivity and specificity to identify lymphoid-PID diagnostic groups from non-PID disease controls in relation to the number of defective lymphoid populations detected with PIDOT.

	Lymphoid-PID (n = 283)	(S)CID, ID and CVID (n = 83)			
		(S)CID (n = 27)	ID (n = 9)	CVID (n = 47)	Total (n = 83)
Defective cell count ratio (95% CI) ^$^	2.87^***^ (2.02-4.07)	6.26^***^ (3.58-11.48)	6.72^***^ (2.97-17.85)	5.01^***^ (3.49-7.27)	5.61^***^ (4.06-7.79)
Sensitivity	61%^1^	70%^2^	89%^2^	87%^2^	82%^2^
Specificity	60%^#,1^	81%^##,2^	81%^##,2^	81%^##,2^	81%^##,2^
LR+	1.55^1^	3.71^2^	4.69^2^	4.60^2^	4.32^2^
cvAUC (95% CI)	0.633 (0.572-0.694)	0.799 (0.645-0.954)	0.935 (0.853-1.000)	0.873 (0.783-0.964)	0.857 (0.780-0.932)

^$^ ***p < 0.001, negative binomial regression, results expressed as ratio (estimated mean number of defective cell counts in lymphoid-PID on estimated mean number of defective cell counts in non-PID disease controls). ^#^ Calculated on non-lymphoid PIDs and non-PID DCs (n = 151). ^##^ Calculated on the non-PID disease controls (n = 116). ^1^ Sensitivity determined at ≥1 lymphoid defect(s), ^2^ Sensitivity determined at ≥2 lymphoid defect(s). cvAUC, cross-validated area under the curve; CI, confidence interval; CVID, common variable immunodeficiency; DCs, disease controls; LR+, positive likelihood ratio; PID, primary immunodeficiency; PAD, predominantly antibody deficiencies; (S)CID, severe combined immunodeficiency; ID, immune dysregulation disorder.

#### 3.3.2 Detailed characterization of lymphoid subset defects

The frequency of patients with lymphoid defects and corresponding LR+, found for each patient group, are shown in **Table 3** and **Figure 2C**. When comparing all lymphoid-PID patients with non-PID DCs, significantly higher percentages of patients with lymphoid defects were found for total T cells and switched memory B cells, but not for other T- and B-cell subsets analyzed. Interestingly, among lymphoid-PID patients, (S)CID, CVID, and ID cases showed a higher number of altered lymphoid populations in blood compared to non-PID DCs. Thus, CVID patients had increased rates of defective total B-cell, pre-GC, total memory B-cell, unswitched memory B-cell, and switched memory B-cell counts. Besides, an increased number of CVID patients with defective counts for lymphocytes (%) and naive TCD4^+^cell counts were observed. In turn, ID patients showed an increased frequency of lymphoid defects compared to the non-PID DCs for TCD4^+^cell and memory B-cell subsets (including both switched memory and unswitched memory B cells). Unexpectedly, in the ID group, a significantly higher percentage of patients with decreased NK-cell counts was observed. (S)CID patients showed increased percentages of cases with defective counts of all major lymphoid populations including total lymphocytes, total T-cells, TCD4^+^cells, naive TCD4^+^cells, TCD8^+^cells, naive TCD8^+^cells, double-negative (DN) TCRγδ^-^ T cells, total B cells, pre-GCs, and switched memory B cells. In contrast, among the ‘other PAD’ cases, similarly lower frequencies of patients presenting with defective B- and/or T-cell population counts compared to non-PID DCs were observed. Remarkably, for all lymphoid-PID patient groups, no higher percentage of patients with lymphoid defects compared to non-PID DCs was observed for central memory, effector memory, effector terminal differentiated TCD4^+^ and TCD8^+^cells, and TCRγδ^+^ T cells. In the non-lymphoid-PID cohort, significantly higher percentages of patients with lymphoid defects were found for total memory B-cell counts compared to non-PID DCs.

**Table 3:** Comparison of percentage of patients with numerical lymphoid defects identified per lymphoid subpopulation for PID (sub)groups with the non-PID disease controls.

	Lymphoid-PID (n = 283)	Lymphoid-PID subgroups					Non-lymphoid-PID(n = 35)	Non-PID DC(n = 116)
		(S)CID(n = 27)	ID(n = 9)		CVID(n = 47)	Other PAD(n = 200)	
% Lymphocytes	12.4% (3.65)	22.2% (6.53)	11.1% (3.26)	23.4%^***^ **(6.88)**		8.5% (2.50)	14.3% (4.21)	3.4%
Lymphocytes/µL	12.0%(3.54)	33.3%^**^ **(9.79)**	22.2% (6.53)	19.1% (5.62)		7.0% (2.06)	8.6% (2.53)	3.4%
B cells/µL	8.1% (∞)	22.2%^***^ **(∞)**	0.0% (∞)	23.4%^***^ **(∞)**		3.0% (∞)	5.7% (∞)	0.0%
T cells/µL	15.5%^**^ **(4.56)**	51.9%^***^ **(15.26)**	33.3% (9.79)	17.0% (5.00)		9.5% (2.79)	5.7% (1.68)	3.4%
TCD4^+^cells/µL	11.0% (6.47)	48.1%^***^ **(28.29)**	33.3%^***^ **(19.59)**	12.8% (7.53)		4.5% (2.65)	5.7% (3.35)	1.7%
TCD8^+^cells/µL	11.7% (4.50)	33.3%^***^ **(12.81)**	22.2% (8.54)	10.6% (4.08)		8.5% (3.27)	8.6% (3.31)	2.6%
PreGC B cells/µL	7.4% (∞)	18.5%^***^ (∞)	0.0% (∞)	14.9%^***^ (∞)		4.5% (∞)	0.0% (∞)	0.0%
Unswitched B cells/µL	10.2% (3.00)	18.5% (5.44)	55.6%^***^ **(16.35)**	31.9%^***^ **(9.38)**		2.0% (0.59)	20.0% (5.88)	3.4%
Switched B cells/µL	23.3%^***^ **(4.48)**	33.3%^***^ **(6.40)**	77.8%^***^ **(14.96)**	70.2%^***^ **(13.50)**		8.5% (1.63)	20.0% (3.85)	5.2%
Total memory B-cells/µL	15.9% (3.70)	25.9%^**^ **(6.02)**	44.4%^***^ **(10.33)**	46.8%^***^ **(10.88)**		6.0% (1.40)	22.9%^**^ **(5.33)**	4.3%
Naive TCD4^+^cells/µL	8.5% (5.00)	40.7%^***^ **(23.94)**	11.1% (6.53)	17.0%^**^ **(10.00)**		2.0% (1.18)	0.0% (0.00)	1.7%
CM TCD4^+^cells/µL	14.8% (1.56)	29.6% (3.12)	33.3% (3.51)	17.0% (1.79)		11.5% (1.21)	11.4% (1.20)	9.5%
EM TCD4^+^cells/µL	12.4% (1.59)	11.1% (1.42)	33.3% (4.27)	19.1% (2.45)		10.0% (1.28)	11.4% (1.46)	7.8%
Effector TD TCD4^+^cells/µL	0.0% (∞)	0.0% (∞)	0.0% (∞)	0.0% (∞)		0.0% (∞)	2.9% (∞)	0.0%
Naive TCD8^+^cells/µL	10.2% (1.96)	37.0%^***^ **(7.12)**	22.2% (4.27)	12.8% (2.46)		5.5% (1.06)	2.9% (0.56)	5.2%
CM TCD8^+^cells	6.7% (1.29)	11.1% (2.13)	11.1% (2.13)	10.6% (2.04)		5.0% (0.96)	11.4% (2.19)	5.2%
EM TCD8^+^cells/µL	4.6% (1.77)	0.0% (0.00)	0.0% (0.00)	6.4% (2.46)		5.0% (1.92)	5.7% (2.19)	2.6%
Effector TD TCD27^+^8^+^cells/µL	3.2% (3.56)	0.0% (0.00)	11.1% (12.33)	0.0% (0.00)		4.0% (4.44)	2.9% (3.22)	0.9%
Effector TD CD8^+^cells/µL	3.9% (2.29)	0.0% (0.00)	11.1% (6.52)	2.1% (1.24)		4.5% (2.65)	5.7% (3.35)	1.7%
DNT TCRγδ^-^ T cells/µL	4.9% (5.44)	22.2%^***^ **(24.67)**	22.2% (24.67)	2.1% (2.33)		2.5% (2.78)	2.9% (3.22)	0.9%
TCRγδ^+^ T cells/µL	14.1% (1.64)	25.9% (3.01)	22.2% (2.58)	12.8% (1.49)		12.5% (1.45)	22.9% (2.66)	8.6%
NK cells/µL	21.6% (2.27)	22.2% (2.34)	66.7%^***^ **(7.02)**	36.2% (3.81)		16.0% (0.68)	20.0% (2.11)	9.5%

Results expressed as percentage of cases with findings for the specific PIDOT subpopulations below the lower limit of normal values. The positive likelihood ratios are presented in parentheses. ***< 0.001, **p < 0.01, chi-squared test. DC, disease controls; PAD, predominantly antibody deficiency; CID, combined immunodeficiency; ID, immune dysregulation; CVID, common variable immunodeficiency; SCID, severe combined immunodeficiencies; CM, central memory; EM, effector memory; PreGC, pre-germinal center; TD, terminal differentiated; DNT, double-negative T cells; NK, natural killer cells. Bold text is to highlight the values which are significantly different from the non-PID DC group.

### 3.4 PIDOT decision-tree algorithm

**Figure 3:**
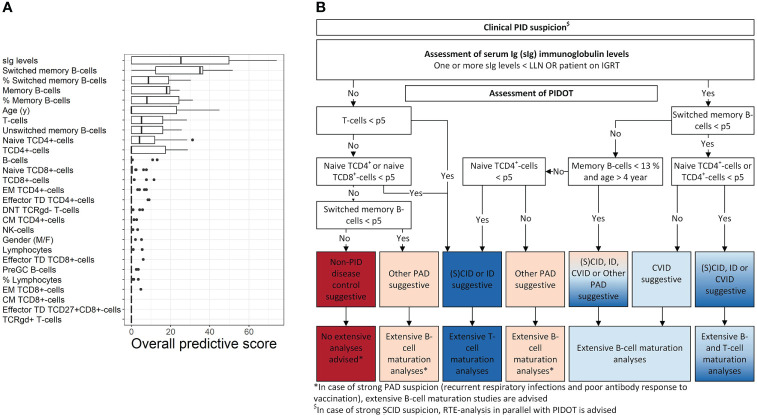
PIDOT decision-tree algorithm. Using supervised machine learning, pruned decision trees were trained with 24 FCM-based PIDOT features supplemented with age- sex- and/or the age-adjusted serum Ig levels (including IgG, IgG_2_, IgG_3_, IgA, and IgM, in case no IGRT at time of basic immunological screening) of non-PID disease controls and lymphoid-PID patient samples. **(A)** Box plots of the overall importance score values of the individual (discriminating) features for distinguishing of lymphoid-PID (subtypes) vs. non-PID disease controls. Only the importance score values of the pruned decision trees with cross-validated balanced accuracy ≥80% are shown. Adding models with a lower balanced accuracy did not impact the importance score values of the individual features. The boundaries of the box plots represent the 25th and 75th percentiles. A black vertical line indicates the median. **(B)** Decision-tree algorithm for early PID screening of lymphoid-PID to guide (more) extensive flow cytometry. CM, central memory; non-PID DCs, non-PID disease controls; DNT, double-negative T cells; EM, effector memory; CID, combined immunodeficiency; CVID, common variable immunodeficiency; sIg, serum immunoglobulin; ID: immune dysregulation; NK, natural killer; PAD, predominantly antibody deficiency; SCID, severe combined immunodeficiency; TCR, T-cell receptor; TD, terminal differentiated.

First, supervised machine learning was used to model two-class pruned decision trees to distinguish between lymphoid-PID (groups) and non-PID DCs. An overview of the overall importance score values of the individual features observed across these models is shown in **Figure 3A**. The most discriminative features in our study cohort were sIg levels, age (y) together with the absolute counts in blood of total memory (and %), unswitched memory and switched memory B cells (and %), total T cells, TCD4^+^cells, and naive TCD4^+^cells. The absolute counts of total T cells, TCD4^+^cells, naive TCD4^+^cells, and total memory and switched memory B cells (and %) were found most predictive to distinguish (S)CID and ID from non-PID DCs. In turn, sIg levels and the absolute counts of total, unswitched, and, switched memory B cells (and % switched memory B cells) were the most predictive features to distinguish CVID from non-PID DCs. In contrast, sIg levels were found to be the only predictive feature to distinguish ‘other PAD’ from non-PID DCs.

Next, the top 10 most predictive features overall were used as input features in the rpart package in R to design the final proposed decision-tree algorithm shown in **Figure 3B**. Thus, our proposed decision-tree algorithm comprises sIg-level assessment of patients with PID suspicion in a first step, followed by PIDOT assessment to guide subsequent (more) extensive FCM-based analyses. More details in what context additional FCM-based analyses are recommended by the here proposed decision-tree algorithm are shown in **Figure 3B**. The sensitivity of the here proposed decision-tree algorithm to correctly suggest lymphoid-PID was 86% with a LR+ of 4.78 (**Supplementary Figure 5**). In detail, the proposed decision-tree algorithm identified all CVID patients as suggestive for lymphoid-PID [7/47 (15%) of these patients were identified as suggestive for ‘other PAD’ due to both normal (switched) memory B-cell and naive TCD4^+^cell counts]. In turn, in the (S)CID and ID patient groups, 32/36 (89%) patients were identified as suggestive for lymphoid-PID [5/36 (14%) and 1/36 (3%) of these patients were defined as suspicious of CVID or ‘other PAD’ due to normal (naive) TCD4^+^cell counts, respectively]. In the ‘other PAD’ patient group, 179/200 (90%) patients were positively identified as suggestive for lymphoid-PID and 10% (n = 19/200) of the ‘other PAD’ patients were identified as being suggestive for (S)CID, ID, or CVID. The overall false negative rate of the proposed decision-tree algorithm was 9% (n = 25/283): 17/25 (68%) patients with SPAD, 4/25 (16%) patients with THI, 2/25 (8%) patients with DiGeorge syndrome (*22q11 del*), 1/25 (4%) patients with hyper-IgE syndrome (*IL6ST*), and 1/29 (4%) patients with CHARGE syndrome (*CHD7*). In addition, the decision-tree algorithm was challenged with the non-lymphoid-PID samples. Of this latter group, 21/35 (60%) were labeled as being suggestive for non-PID DC, whereas the remaining 14/35 (40%) patients were identified as suggestive for lymphoid-PID [6% as (S)CID or ID, 3% as CVID, and 31% as ‘other PAD’].

Finally, we applied the previously published EuroFlow PID screening and classification algorithm, for guiding more extensive follow-up FCM-based T- and/or B-cell analyses [‘classical (EuroFlow) approach’] and compared this approach with the criteria as defined by the here proposed decision-tree algorithm [‘decision-tree approach’] in **Table 4**. The approaches showed no significant differences for percentage of cases per PID diagnostic subtype for whom follow-up FCM-based B-cell analysis is advised. In contrast, the ‘decision-tree approach’ had a significantly lower percentage of cases for whom follow-up FCM-based T-cell analyses is advised compared to ‘the classical (EuroFlow) approach’ for all patient groups except (S)CID and ID.

**Table 4:** Detailed follow-up FCM-based T- and/or B-cell analyses as guided by the EuroFlow PID algorithm compared to the proposed decision-tree algorithm.

	EuroFlow PID algorithm			Proposed decision-tree algorithm		
	Detailed B-cell analysis advised	Detailed T-cell analysis advised	No extra FCM analysis advised	Detailed B-cell analysis advised	Detailed T-cell analysis advised	No extra FCM analysis advised
(S)CID and ID (n = 36)	53%	69%	19%	56%^ns^	56%^ns^	14%^ns^
CVID (n = 47)	79%	57%	13%	83%^ns^	17%^***^	15%^ns^
Other PAD (n = 200)	14%	37%	37%	13%^ns^	4%^***^	88%^***^
Non-lymphoid PID (n = 35)	29%	37%	57%	3%^ns^	3%^**^	91%^**^
Non-PID DC (n = 116)	6%	27%	71%	3%^ns^	9%^***^	90%^***^

The percentage of cases per PID diagnostic subgroup for whom additional testing is (not) advised. ***p < 0.001, **p < 0.01, ns: non-significant, chi-squared test. DC, disease controls; PAD, predominantly antibody deficiency; CID, combined immunodeficiency; ID, immune dysregulation; CVID, common variable immunodeficiency; SCID, severe combined immunodeficiencies.

## 4 Discussion

At present, FCM plays a critical role in the diagnostic workup of patients suspicious of PID. Despite this, routine use of FCM in PID diagnostic screening still faces important challenges, particularly with regard to standardization. In recent years, the EuroFlow Consortium has taken major steps to standardize multiparameter FCM as part of the PID diagnostic workup, by designing PIDOT and establishing SOPs for sample preparation (31). In this study, we report for the first time on the performance of PIDOT in a large series of consecutive patients suspicious of PID, studied at a single reference center including both pediatric and adult patients. In addition, we propose an optimized decision-tree algorithm to guide subsequent more extensive FCM analyses in order to enhance the clinical performance and utility of the EuroFlow PID diagnostic approach in routine patient care.

Overall, our results showed that more defective counts for various lymphoid populations together with an increased frequency of alterations for several lymphoid cell populations are found among patients with various diagnostic subtypes of lymphoid-PID [e.g., (S)CID, ID, and CVID] compared to patients in whom PID is excluded. In this regard, the presence of numerical defects involving at least two lymphoid cell populations provided the highest accuracy in terms of both sensitivity and specificity, for discrimination between (S)CID, ID, and CVID and non-PID disease controls. The lymphoid cell populations found to be most discriminative included the absolute counts of total lymphocytes (and %), total B cells, pre-GC B cells, switched memory B cells, unswitched memory B cells, total memory B cells, total T cells, TCD4^+^cells, TCD8^+^cells, naive TCD4^+^cells, naive TCD8^+^cells, DN TCRγδ^-^ T cells, and NK cells.

In contrast, as might be expected, our results showed that PIDOT was not able to clear-cut discriminate patients with ‘other PAD’ from non-PID DCs, as the majority of these patients did not show any defective lymphoid counts. These findings together with the relatively high frequency of ‘other PAD’ patients represented in the study cohort obviously had a negative impact on the overall sensitivity (0.61) and LR+ (1.55) for the wider spectrum of lymphoid-PID that were included in the study. Our findings, showing limited or no T- and B-cell (maturation-associated) defects among patients classified here as ‘other PAD’, are similar to previous FCM-based studies on PAD patients. These studies show that >70% of patients with unclassified idiopathic primary hypogammaglobulinemia or isolated IgA or IgG subclass deficiencies have normal T- and B-cell patterns compared to their age-matched healthy control groups (35, 49–52). Thus, for these cases the EuroFlow IgH-isotype tube, providing more detailed dissection of the B-cell and particularly the plasma-cell (PC) compartments, would be appropriate, as recommended by the EuroFlow Consortium, since it has been shown to reveal one or more B-cell defect(s) in every patient with IgA or IgG/A deficiency (e.g., PC counts are decreased in 49% to 90% of these patients) (30, 35). Therefore, according to the here proposed decision-tree algorithm and the previously published EuroFlow diagnostic PID algorithm, it is recommended to perform more extensive FCM-based analyses of the B-cell and PC maturation-associated compartments in blood, in case of strong suspicion of a PAD diagnosis, even when no lymphoid cell defects are found with PIDOT, to improve the diagnostic accuracy of FCM in this (numerous) group of PAD patients (30). Lastly, as might be expected, PIDOT was not able to clear-cut discriminate non-lymphoid-PID patients from non-PID disease controls based on the total number of defective lymphoid counts found per patient and the percentage of patients presenting with defects in the distinct T- and B-cell populations. Nonetheless, heterogeneous patterns of lymphoid defects were observed with PIDOT in almost half of these non-lymphoid-PID patients, which is in line with previous findings by van der Burg et al. (2019) (31).

Based on the PIDOT features and some additional features such as serum Ig levels and age, we designed a decision-tree algorithm for broad use in clinical practice aiming at more efficient guidance of (more) extensive FCM workup after PIDOT analysis and, thereby also, more efficient diagnosis of lymphoid-PID. From all parameters investigated using supervised machine learning, serum Ig levels; the absolute counts in blood of the total T cells, TCD4^+^cells, naive TCD4^+^cells, total B cells, and total memory (and %) and switched memory B cells (and %); and age emerged as the most discriminating parameters (26, 53–55). Based on ROC analysis, the decision-tree algorithm here proposed showed a high (86%) sensitivity and (82%) specificity (LR+ of 4.78), associated with an increased cost efficiency and decreased workload in the diagnostic workup of PIDs, as compared to the classical EuroFlow PID diagnostic algorithm, as the percentage of patients in whom additional FCM-based T-cell analyses would be advised was significantly lower with our model, particularly in the ‘non-lymphoid-PID’ and non-PID DC groups. Importantly, our decision-tree algorithm is only designed for guidance to optimize the clinical utility of PIDOT and the subsequent FCM analyses, and therefore it does not exclude the need for further FCM-based analyses in case of strong clinical suspicion of SCID/PAD, including parallel analysis of the EuroFlow IgH-isotype tube recommended previously (and also above) in case of ‘other PAD’ (30). Of note, according to the ESID criteria, specific lymphoid population defects that are mandatory for PID screening included the counts of total lymphocytes, total B-cells, switched memory B cells, total T cells, TCD4^+^ and TCD8^+^cells, naive TCD4^+^cells, naive TCD8^+^cells, and NK cells, all of which are evaluated with PIDOT (12). Despite all the above, it should be noted that the multiparameter FCM immunophenotyping approach here evaluated should not to be used as a stand-alone diagnostic tool for lymphoid-PID and it should always be integrated with other complementary diagnostic tools (such as functional and genetic assays) for optimal PID diagnostics. For instance, in several PID subtypes, which might present with no or minimal lymphoid defects and/or preserved or borderline serum Ig levels, such as in case of some partial DiGeorge syndrome (DGS) patients, a subset of ALPS patients, Nijmegen breakage syndrome patients, and ataxia telangiectasia patients at a young age, functional and genetic tests are more informative (30). In our study, a fraction of the patients identified as suggestive for non-PID DC based on PIDOT and the here proposed diagnostic algorithm were in fact SPAD (19%) and DGS (2%) patients in whom further FCM studies with, e.g., the EuroFlow IgH-isotype tube and/or genetic analyses, should be performed.

In this study, we report on our experience with PIDOT in a single tertiary care center focusing on the FCM diagnostic workup of patients with PID suspicion, to gain insight in how PIDOT, as well as the here proposed decision-tree algorithm, would perform in daily clinical practice, and how it would help guide laboratory testing for more efficient diagnostics. However, the value of such a study is limited by the number of patients with rare PID diagnostic subtypes and that are underrepresented or even missing in our patient cohort. Thus, further evaluation and validation of PIDOT in combination with the here proposed decision-tree algorithm in larger (e.g., multicentric) cohorts of patients that include rare diagnostic subtypes of PID are needed to further support the clinical utility of the proposed decision-tree algorithm.

In summary, our findings confirm that PIDOT is a powerful tool for PID diagnostic screening, particularly in (S)CID, ID, and CVID patients, against a background of non-lymphoid-PID and non-PID disease controls. Furthermore, its combination with the decision-tree algorithm here proposed provides an adequate and cost-effective guidance for subsequent (more) extensive FCM analyses, by integration of PIDOT results with data on patient age and serum Ig levels. These results and tools will support wide implementation of PIDOT and the supplementary EuroFlow PID tubes in other PID diagnostic centers for standardized FCM diagnostics in PID, complementary to functional and genetic assays. Wider application of the EuroFlow lymphoid-PID approach will contribute to improve PID diagnostics and will expand the options for data exchange and integration between different PID centers, which is of utmost importance in the context of rare diseases such as PID.

## Data availability statement

The raw data supporting the conclusions of this article will be made available by the authors, without undue reservation.

## Ethics statement

This study was reviewed and approved by Ghent University Hospital Ethics Committee, Ghent, Belgium. The patients/participants provided their written informed consent to participate in this study.

## Author contributions

CB, MH, FH, JP, AO, MP-A, MvdB, JD, and JP contributed to the conception and design of the study. JN, first author, generated the results and drafted the initial manuscript under the supervision of MH, FH, and CB. JN and MB performed data acquisition and data analysis. AE, SG, YS, and SD supported statistical methods and the computational learning analysis. FH, BL, and TK provided the clinical data. All authors contributed to the article and approved the submitted version.

## Funding

CB, FH and XB are supported by an FWO TBM grant (Research Foundation – Flanders, T000119N). JN is a PhD fellow of the Research Foundation Flanders (FWO, 11L2822N). FH is funded by a University Research Grant (BOF-University Ghent) and Center for Primary Immune deficiency Ghent is funded by Jeffrey Modell Foundation. This project received funding within the Grand Challenges Program of VIB. This VIB Program received support from the Flemish Government under the Management Agreement 2017–2021 (VR 2016 2312 Doc.1521/4).

## Acknowledgments

The authors would like to thank the patients and their families and the healthy blood donors for participating in the study. We also gratefully acknowledge Pauline Breughe and Jana De Wolf for their technical support and their contribution in PIDOT data analyses. We would like to gratefully thank our clinical nurse Karlien Claes for the excellent clinical support and assistance in blood sampling. This study was supported by the EuroFlow Consortium. The EuroFlow Consortium received support from the FP6-2004-LIFESCIHEALTH-5 program of the European Commission (grant LSHB-CT-2006-018708) as Specific Targeted Research Project (STREP). The EuroFlow Consortium is part of the European Scientific Foundation for Haemato-Oncology (ESLHO), a Scientific Working Group (SWG) of the European Haematology Association (EHA). All members of the EuroFlow PID Work package (chaired by MvdB) are greatly acknowledged for their contribution, critical insights, and helpful discussions.

## Conflict of interest

JD and AO each report being two of the inventors on the EuroFlow-owned patent PCT/NL 2015/050762 (diagnosis of primary immunodeficiencies). The Infinicyt software is based on intellectual property (IP) of the EuroFlow laboratories (University of Salamanca in Spain and Federal University of Rio de Janeiro in Brazil) and the scientific input of other EuroFlow members. All abovementioned intellectual property and related patents are licensed to Cytognos (Salamanca, ES) and BD Biosciences (San José, CA), for which companies pay royalties to the EuroFlow Consortium. These royalties are exclusively used for continuation of the EuroFlow collaboration and sustainability of the EuroFlow Consortium. JD and AO report an Educational Services Agreement from BD Biosciences and a Scientific Advisory Agreement from Cytognos; all related fees and honoraria are for the involved university departments at Leiden University Medical Center and University of Salamanca.

The remaining authors declare that the research was conducted in the absence of any commercial or financial relationships that could be construed as a potential conflict of interest.

## Publisher’s note

All claims expressed in this article are solely those of the authors and do not necessarily represent those of their affiliated organizations, or those of the publisher, the editors and the reviewers. Any product that may be evaluated in this article, or claim that may be made by its manufacturer, is not guaranteed or endorsed by the publisher.
