# Supplementary material for: The Euroflow PID Orientation Tube in the diagnostic workup of primary immunodeficiency: Daily practice performance in a tertiary university hospital
Source: Front Immunol. 2022 Sep 13;13:937738. doi: 10.3389/fimmu.2022.937738 (PMC9513319; doi:10.3389/fimmu.2022.937738)
Supplement: Supplementary file 1 [file DataSheet_1.pdf]

Supplementary Material

1 Supplementary Methods

Staining procedure, Instrument settings and Data acquisition

PB samples were collected in BD Vacutainer tubes containing K_2EDTA (Becton/Dickinson, San Jose, CA). Non-nucleated red cells were lysed prior to staining, strictly following the EuroFlow bulk lyse SOP. Subsequently, a stain- wash protocol was performed. Thus, the remaining cell pellet in a volume of 100µL, was stained for 30 minutes in the dark (room temperature [RT]) with the EuroFlow PIDOT monoclonal antibody combination (both lyophilized kit [CYT-PIDOT, Cytognos SL, Salamanca, Spain] and liquid format are used). Afterwards, 2mL of BD FACS™ Lysing solution [Becton/Dickinson Biosciences (BD)], diluted 1/10 (v/v) in distilled water, was added to the cell suspension followed by incubation for another 10 minutes at RT in the dark. Afterwards, cells were washed and finally the cell pellet was re-suspended in 300µL of washing buffer. Staining and data acquisition of all samples were performed within 24h after blood collection. Data were acquired on BD FACSCanto™II and BD FACSLyric™ flow cytometers (Becton/Dickinson, San Jose, CA). Instrument settings and data acquisition were performed according to the EuroFlow guidelines available at www.EuroFlow.org. Standard instrument settings were monitored by BD™ Cytometer Setup and Tracking (CS&T) beads (BD) and eight-peak Rainbow bead calibration particles for FACSCanto only (Spherotech, Lake Forest, IL). For each sample at least 10^6 total events were acquired. As per EuroFlow standard instrument setup and calibration SOPs, further manual compensation for optimization of measurements of individual samples was not required.

PIDOT gating, data analysis and calculations

Gating of the lymphoid populations was performed after excluding debris and cell doublets based on sideward light scatter area (SSC-A)/forward light scatter area (FSC-A) and FSC Height (FSC-H)/FSC-A bivariate dotplots, respectively. B-cells were identified based on their unique $CD45^{hi}CD19^{+}CD3^{-}$ $CD45RA^{+}$ phenotype and $FSC^{lo}SSC^{lo}$ characteristics. CD27, IgM and IgD were used for further identification of the B-cell subpopulations: switched and unswitched memory B-cells. T-cells were identified based on a $CD45^{hi}CD3^{+}$ and $FSC^{lo}SSC^{lo}$ phenotype. After gating $TCR\gamma\delta^{+}$ T-cells, the $TCD4^{+}$-, $TCD8^{+}$- and $TCD4^{-}CD8^{-}TCR\gamma\delta^{-}$-cell populations were identified. Maturation stages of

$TCD4^+$- and $TCD8^+TCR\gamma\delta^-$-cells were identified based on CD27 and CD45RA expression levels. NK-cells were defined as $CD45^{hi}CD19^-CD3^-CD16\&CD56^{hi}CD45RA^{loto+}FSC\text{-}A^{lo}SSC\text{-}A^{lo}$ cells (**Supplementary Table 3**). After manual gating, the absolute lymphocyte cell count (/µL of blood) was calculated, using the patient's lymphocyte counts obtained from the Sysmex XE-5000 hematology analyzer for each identified lymphocyte population (n=21). Absolute lymphocyte cell counts of 21 lymphoid populations and % lymphocytes were compared to the EuroFlow age-matched p5-p95 reference ranges as defined based on a series of 250 healthy controls aged between 0 days [neonatal] and 89 years divided into 14 different age groups) to identify lymphoid defects in each patients. To adjust for age, the quantitative absolute cell counts (/µL) per lymphocyte subpopulation were converted into categorical data: 8 categories based on the percentile ranges [(1) <p5, (2) p5-p10, (3) p10-p25, (4) p25-p50, (5) p50-p75, (6) p75-p90 (7) and (8) >p95] or 3 categories only [(1) <p5, (2) p5-p95, (3) >p95] using the EuroFlow age-matched p5-p95 reference ranges.

Design of a decision-tree algorithm

Supervised machine learning was performed using Recursive Partitioning ("rpart"package v4.1.15) in R (v4.0.3) followed by 10-fold cross-validation ("ipred" package v0.9.12). First, a selection of the most predictive features to distinguish between lymphoid-PID (groups) and non-PID disease controls from the input dataset (described in Section 2.4) was performed. In total, fifty two-class pruned decision-trees were trained with different endpoints and combinations of input variables. The selected endpoints of these models were (1) (S)CID and ID, (2) CVID, (3) 'other' PAD, (4) lymphoid-PID with 'other' PAD excluded and (5) all lymphoid-PID patients versus the non-PID disease controls. The selected combinations of input variables were (1) age-adjusted absolute cell counts (/µL) based on three or eight categories (n=21) supplemented with % total memory and switched memory B-cells and % lymphocytes, (2) age-adjusted absolute cell counts (/µL) based on three or eight categories (see above) without the less abundant T-cell populations (n=16) supplemented with % total memory and switched memory B-cells and % lymphocytes and (3) previous combinations supplemented with age and gender and/or serum Ig levels. The pruned decision-trees with cross-validated balanced accuracy ≥80% were retained for further assessment (n=36/50 with an average balanced accuracy of 85%). Subsequent, the input features were ranked according to their overall importance score values in these models. The final decision-tree algorithm was designed for lymphoid-PID screening and guidance for subsequent more extensive follow-up FCM-based analyses as follows. First, the input feature with the highest overall importance value was selected as the first split in the decision-tree algorithm and used to

subdivide the study cohort of lymphoid-PID and non-PID disease controls into two groups. Afterwards, these two groups were used to train (multi-class) decision-trees with different endpoints and the remaining top ranked features as input features. The selected endpoints of these models were (1) lymphoid PID vs. non-PID DCs, (2) (S)CID, ID or CVID vs 'other PAD' vs. non-PID DCs, (3) (S)CID, ID vs. CVID vs 'other PAD' vs. non-PID DCs. The (multiclass) decision-trees with highest cross-validated balanced accuracy in each group were retained and further optimized based on expert opinion: (1) the naive $TCD8^{+}$cell count was added to the supervised decision-tree model to avert false negativity for CD8 deficient patients (because no patient with this phenotype was included in our dataset), (2) the naive $TCD4^{+}$cell counts was also added to the supervised decision-tree model, after switched-memory B-cells assessment in case of decreased serum Ig levels, to improve detection of patients with late onset combined immunodeficiency (LOCID), a subset within CVID. Finally, results were combined into one final decision-tree algorithm. The predicted probability for lymphoid-PID retrieved from the decision-tree algorithm of each lymphoid-PID patient and non-PID DC were used to evaluate the model performance as described in Statistical methods.

Statistical methods

The 10-fold cross-validated Area Under the Curve (cvAUC) of the Receiver Operating Characteristic (ROC) curve was calculated, using the "ROCR" (v1.0-11) and "cvAUC" (v1.1.4) packages in R, to assess performance of PIDOT in relation to the total number of lymphoid population defects and the proposed decision-tree in relation to the probability for lymphoid-PID to distinguish lymphoid-PID from the non-PID DCs. The 95% confidence interval (CI) of cvAUC was computed based on influence curves. Youden's index was calculated to select the cut-off value with the best combination of sensitivity (sens) [sens = true positives/(true positives + false negative)] and specificity (spec) [spec = true negatives/(true negatives + false positives)]. Positive likelihood ratios (LR+) and balanced accuracies (BA) were calculated as follows: [LR+ = sens/(1 – spec); BA = (sens+spec)/2)].

2 Supplementary Figures and Tables

2.1 Supplementary Tables

Supplementary Table 1: Overview of the excluded patients (N=147)

Number of patients	Reason for exclusion
**38**	**Transplantation and/or immune suppressive therapy**
15	Post-HSCT in the context of MDS (n=1), SCID (n=8), bone marrow failure (n=2), acute myeloid leukemia (n=1), β-thalassemia major (n=1), anaplastic T cell lymphoma (n=2)
1	Post gene therapy ADA-SCID
7	(History of) Chemotherapy in the context of Hodgkin lymphoma, AML, burkitt lymphoma, carcinoma
13	Immunosuppressive therapy in context of solid organ transplant, multiple sclerosis, colitis ulcerosa, inflammatory bowel disease, Kaposi form hemangioendothelioma
2	Splenectomy and/or hepatectomy
**28**	**Secondary Immunodeficiency**
2	Thymoma with immunodeficiency (Good's syndrome)
2	Auto-immune cytopenia
2	Protein losing enteropathy
5	Secondary lymphopenia/hypogammaglobulinemia
3	Immune deficiency associated with trisomy 21
5	Hematological malignancies (MDS, Monoclonal B cell lymphocytosis, DLCBCL, Indolent B cell lymphoma, Burkitt lymphoma)
8	Auto-immune diseases (lupus, rheumatoid arthritis, juvenile idiopathic arthritis, graves diseases, hypothyroidism)
1	Congenital surfactant type C
**28**	**Technical issues PIDOT**
6	No interpretation of PIDOT possible due to suspicion of abnormal CD45 splicing
2	Technical issue with datafile
20	No white blood cell count was determined
**53**	**No/insufficient clinical information in patient clinical record (e.g. external lab request)**

Abbreviations: ADA, Adenosine deaminase deficiency; AML, Acute myeloid leukemia; DLCBL, Diffuse large cell B cell lymphoma; MDS, Myelodysplastic syndrome, HSCT, Hematological stem cell transplantation; SCID, Severe combined immunodeficiency

Supplementary Table 2: Study population demographics

		Number (male/female)	Median Age[1] (min-max)	IGRT[1] (% of patients)
Lymphoid-PID	(S)CID/CID	27 (17/10)	8 (0.1-37)	52%
	ID	9 (4/5)	15 (4-49)	11%
	CVID	47 (26/21)	36 (4-77)	60%
	Other PAD	200 (91/109)	19 (0.1-80)	26%
Non-lymphoid PID		35 (14/21)	5 (0.8-19)	11%
Non-PID DC		116 (75/41)	2 (0.1-72)	0%

[1]Recorded at time of PIDOT screening. **Abbreviations:** ID, immune dysregulation disorder, Non-PID DC, non-PID disease controls; CID, Combined immunodeficiencies; IGRT, Immunoglobulin replacement therapy; PAD, Predominantly antibody deficiencies; SCID, Severe combined immunodeficiency; CVID, Common Variable immunodeficiency; PID, primary immunodeficiency

Supplementary Table 3: Manual Gating strategy for the identification of lymphoid populations in blood according to the EuroFlow guidelines for analysis of blood samples stained with PIDOT.

Population	Gating strategy
B-cells	FSC^{lo} $SSC^{lo}CD45^{hi}CD19^{+}CD3^{-}CD45RA^{+}$
• Pre-germinal center B-cells	$CD27^{-}IgD^{+}IgM^{+}$
• Post-germinal center B-cells/plasmacells (MBC/PC)	
▪ Unswitched MBC/PC	$IgD^{+}IgM^{+}CD27^{+}$
▪ Switched MBC/PC	$IgD^{-}IgM^{-}CD27^{-\text{ to }+}$
▪ $IgD^{+}IgM^{-}$ post-GC	$IgD^{+}IgM^{-}CD27^{+}$
T-cells	FSC^{lo} $SSC^{lo}CD45^{hi}CD3^{+}CD19^{-}$ $CD16\&CD56^{-\text{ to lo}}$
• $TCR\gamma\delta^{+}$ T-cells	$TCR\gamma\delta^{+}CD4^{-}CD8^{-\text{ to lo}}$
• $TCR\gamma\delta^{-}$ $CD4^{-}CD8^{-}$ T-cells	$TCR\gamma\delta^{-}CD4^{-}CD8^{-\text{ to lo}}$
• $TCD4^{+}$-cells	$TCR\gamma\delta^{-}CD4^{+}CD8^{-}$
▪ Naive $TCD4^{+}$ -cells	$CD27^{+}CD45RA^{+}$
▪ Central memory $TCD4^{+}$-cells	$CD27^{+}CD45RA^{-}$
▪ Effector memory $TCD4^{+}$-cells	$CD27^{-}CD45RA^{-}$
▪ Terminal effector $TCD4^{+}$-cells	$CD27^{-}CD45RA^{+}$
• $CD8^{+}$ T-cells	$TCR\gamma\delta^{-}CD4^{-}CD8^{+}$
▪ Naive $TCD8^{+}$ -cells	$CD27^{+}CD45RA^{+}$
▪ Central memory $TCD8^{+}$ -cells	$CD27^{+}CD45RA^{-}$
▪ Effector memory $TCD8^{+}$ -cells	$CD27^{-}CD45RA^{-}$
▪ Effector CD27+ $TCD8^{+}$ -cells	$CD27^{lo}CD45RA^{+}$
▪ Terminal effector $TCD8^{+}$ -cells	$CD27^{-}CD45RA^{+}$
• $TCD4^{+}CD8^{+}$-cells	$TCR\gamma\delta^{-}CD4^{+}CD8^{+}$
Natural Killer cells	$SSC\text{-}A^{lo}FSC\text{-}A^{lo}CD45^{hi}CD19^{-}CD3^{-}$ $CD16\&CD56^{hi}CD45RA^{lo\text{ to }+}$

Supplementary Table 4: The clinical characteristics of the non-PID disease controls (DC) (n=116)

Number of non-PID DC cases	Clinical characteristics for PID suspicion
57 (49 %)	Airway and inflammatory related disorders (incl asthma, bronchial hyperreactivity, obstructive bronchial disease, bronchiectasis, allergy, inflammatory bowel disease)
39 (34 %)	Recurrent viral infections (incl. upper airway infections)
11 (9 %)	Recurrent otitis or otorrhea
10 (9 %)	Recurrent, deep skin or organ abscesses
6 (5 %)	Pneumonia
5 (4 %)	Failure to thrive
5 (4%)	Severe and/or invasive infections (incl. meningitis)
4 (3 %)	Dysmorphia
3 (3 %)	Persistent thrush in mouth or fungal infection on skin or elsewhere
2 (2 %)	Chronic sinusitis
1 (0,9%)	Infection with harmless tuberculosis-like bacteria
1 (0,9%)	Deep-seated infections (incl. septicemia)

Supplementary Table 5: Verification of the EuroFlow reference values using an independent healthy control group (N=68). Numbers represent samples with absolute cell counts outside the 5th and 95th percentile as defined by the EuroFlow database for the lymphoid subpopulations

	**1-23 months (n=5)**		**2-4 years (n=10)**		**5-9 years (n=6)**		**10-17 years (n=6)**		**18-29 years (n=20)**		**30-39 years (n=7)**		**40-49 years (n=5)**		**50-59 years (n=5)**		**60-69 years (n=4)**		**TOTAL (n=68)**	
	<p5	>p95	<p5	>p95	<p5	>p95	<p5	>p95	<p5	>p95	<p5	>p95	<p5	>p95	<p5	>p95	<p5	>p95	<p5	>p95
Lymphocytes	0	1	0	0	0	0	0	0	0	1	0	1	0	0	0	0	0	0	0%	4%
B-cells	0	1	0	0	1	0	0	0	0	0	0	0	0	0	0	0	1	0	3%	1%
T-cells	0	0	0	0	0	0	0	0	0	0	0	0	0	0	1	0	0	0	1%	0%
TCD4^{+}-cells	0	1	1	0	1	0	0	0	1	2	0	1	0	0	0	0	0	0	4%	6%
TCD8^{+}-cells	0	1	0	0	0	0	0	0	0	1	0	1	0	0	0	0	0	0	0%	4%
PreGC B-cells	0	1	0	0	1	0	0	0	0	0	0	0	0	0	0	0	1	0	3%	1%
Unswitched memory B-cells	0	1	0	0	0	0	0	0	0	0	0	0	0	0	0	0	0	0	0%	1%
Switched memory B-cells	0	0	1	0	0	0	0	0	0	0	0	0	0	0	0	0	0	0	1%	0%
Total memory B-cells	0	1	0	0	0	0	0	0	0	0	0	0	0	0	0	0	1	0	1%	1%
Naive TCD4^{+}-cells	0	1	1	0	1	0	0	0	0	1	0	0	0	0	0	0	0	1	3%	4%
CM TCD4^{+}-cells	0	0	1	0	1	0	1	0	1	0	0	1	0	1	0	0	0	1	6%	4%
EM TCD4^{+}-cells	0	1	0	1	0	0	0	0	1	1	0	0	0	1	0	0	1	0	3%	6%
Effector TD TCD4^{+}-cells	0	0	0	1	0	0	0	0	2	1	0	0	0	0	0	0	0	0	3%	3%
Naive TCD8^{+}-cells	0	0	0	0	0	0	0	0	0	0	0	0	1	1	0	1	0	0	1%	3%
CM TCD8^{+}-cells	0	1	0	0	0	0	0	0	0	0	0	1	0	0	0	1	0	0	0%	4%
EM TCD8^{+}-cells	0	0	0	1	0	1	0	2	0	0	0	0	0	0	0	0	0	0	0%	6%
Effector TD TCD27^{+}CD8^{+}-cells	0	0	0	1	0	0	0	0	0	0	0	0	0	0	1	0	0	0	1%	1%
Effector TD TCD8^{+}-cells	0	0	0	1	0	0	0	1	0	1	0	0	0	0	0	0	0	0	0%	4%
DN TCRγδ- T-cells	0	0	1	0	0	0	0	1	0	3	0	1	0	0	0	1	0	0	1%	9%
TCRγδ+ T-cells	0	2	0	0	0	0	0	0	0	1	1	0	0	0	1	0	0	0	3%	4%

Abbreviations: CM, central memory; EM, Effector Memory; PreGC, Pre-Germinal center TD, Terminal Differentiated; DNT, Double Negative T cells, NK, Natural Killer cells; TCR, T cell receptor

2.2 Supplementary Figures

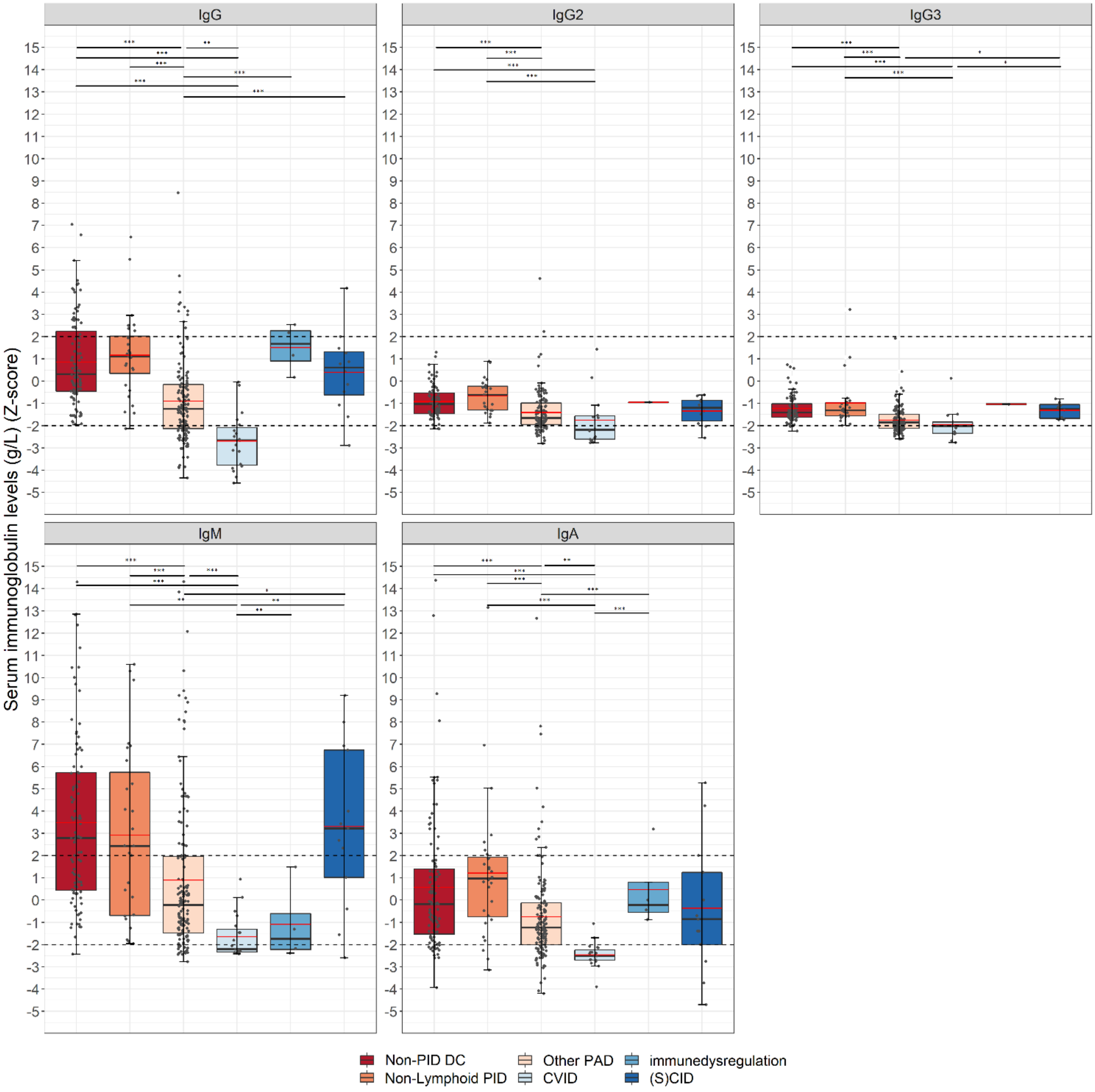

Supplementary Figure 1: Box plots of serum immunoglobulin levels at time of PIDOT analysis. Patients receiving IGRT at time of PIDOT analysis were excluded. Immunoglobulin measurements were expressed as z-scores to adjust for age. Values normal for age have a z-score between -2 and 2 (dotted lines). The boundaries of the box plots represent the 25th and 75th percentiles. A black and red line indicate the median and mean respectively. ***p<0.001,**p<0.01,*p<0.05, Mann-Whitney Rank Sum test. Non-significant p-values are not shown. **Abbreviations:** DC, non-PID disease controls;

PAD, predominantly antibody deficiency; CID, combined immunodeficiency; CVID, common variable immunodeficiency; SCID, severe combined immunodeficiency

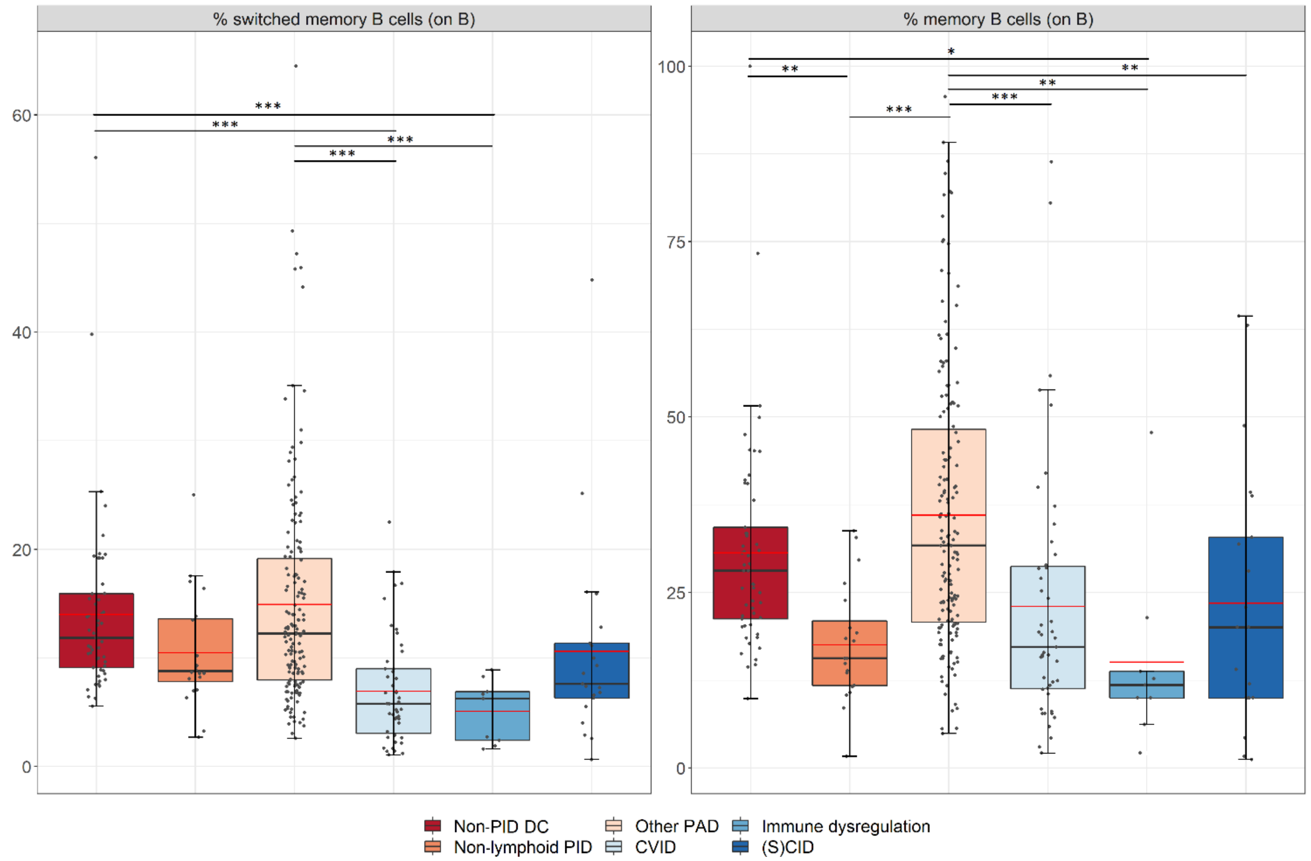

Supplementary Figure S2: Box plots of total memory and switched memory B-cells (% as expressed on the B-cells) measured by the PIDOT. The boundaries of the box plots represent the 25th and 75th percentiles. A black and red line indicate the median and mean respectively. ***p<0.001,**p<0.01,*p<0.05, Mann-Whitney Rank Sum test. Non-significant p-values are not shown. **Abbreviations:** DC, non-PID disease controls; PAD, predominantly antibody deficiency; CID, combined immunodeficiency; CVID, common variable immunodeficiency; SCID, severe combined immunodeficiencies

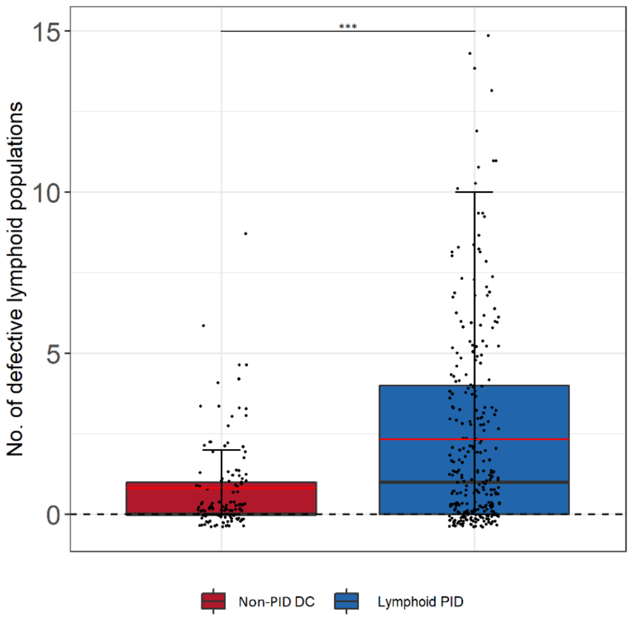

Supplementary Figure 3: Box plots of frequency of total defective lymphoid populations (over the 22 FCM PIDOT variables). The boundaries of the box plots represent the 25th and 75th percentiles. A black and red line indicate the median and mean respectively. ***p<0.001,**p<0.01,*p<0.05, Mann-Whitney Rank Sum test. **Abbreviations:** DC, non-PID disease controls; PID, primary immunodeficiency

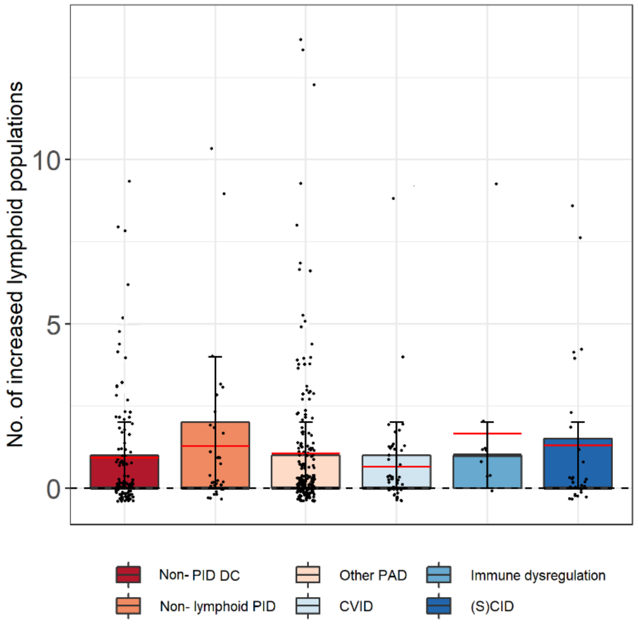

Supplementary Figure 4: Box plots of frequency of total increased cell counts (over the 22 FCM PIDOT variables). The boundaries of the box plots represent the 25th and 75th percentiles. A black and red line indicate the median and mean respectively. No significant adjusted p-values were found

(Mann-Whitney Rank Sum test). **Abbreviations:** DC, non-PID disease controls; PAD, predominantly antibody deficiency; CID, combined immunodeficiency; CVID, common variable immunodeficiency; SCID, severe combined immunodeficiencies

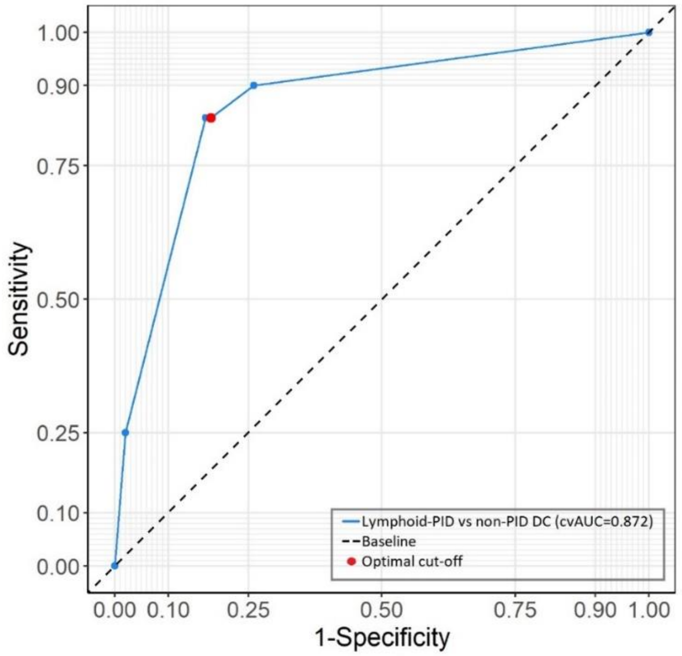

Supplementary Figure 5: Receiver Operating Characteristic (ROC) curve to assess the performance of the decision-tree algorithm in relation to the predicted probabilities for lymphoid-PID. The predicted probability of lymphoid-PID based on the proposed decision-tree algorithm was calculated for each lymphoid-PID patient and non-PID disease control. These probabilities were used to define the 10-fold cross-validated Area Under the Curve (cvAUC) of 0.872.The red dot present the optimal cut-off to identify lymphoid-PID from non-PID DCs (sens = 86%, spec = 82%).
